# Vaccine Effectiveness during Outbreak of COVID-19 Alpha (B.1.1.7) Variant in Men’s Correctional Facility, United States

**DOI:** 10.3201/eid2807.220091

**Published:** 2022-07

**Authors:** Rachel A. Silverman, Alessandro Ceci, Alasdair Cohen, Meagan Helmick, Erica Short, Paige Bordwine, Michael J. Friedlander, Carla V. Finkielstein

**Affiliations:** Virginia Tech, Blacksburg, Virginia, USA (R.A. Silverman, A. Cohen, M.J. Friedlander, C.V. Finkielstein);; Fralin Biomedical Research Institute, Roanoke, Virginia, USA (A. Ceci, M.J. Friedlander, C.V. Finkielstein);; Virginia Department of Health, Richmond, Virginia, USA (M. Helmick, E. Short, P. Bordwine).

**Keywords:** COVID-19, SARS-CoV-2, incarcerated persons, prisons, correctional facilities, vaccine effectiveness, severe acute respiratory syndrome coronavirus 2, viruses, respiratory infections, zoonoses, United States

## Abstract

In April 2021, a COVID-19 outbreak occurred at a correctional facility in rural Virginia, USA. Eighty-four infections were identified among 854 incarcerated persons by facilitywide testing with reverse transcription quantitative PCR (qRT-PCR). We used whole-genome sequencing to link all infections to 2 employees infected with the B.1.1.7α (UK) variant. The relative risk comparing unvaccinated to fully vaccinated persons (mRNA-1273 [Moderna, https://www.modernatx.com]) was 7.8 (95% CI 4.8–12.7), corresponding to a vaccine effectiveness of 87.1% (95% CI 79.0%–92.1%). Average qRT-PCR cycle threshold values were lower, suggesting higher viral loads, among unvaccinated infected than vaccinated cases for the nucleocapsid, envelope, and spike genes. Vaccination was highly effective at preventing SARS-CoV-2 infection in this high-risk setting. This approach can be applied to similar settings to estimate vaccine effectiveness as variants emerge to guide public health strategies during the ongoing pandemic.

Incarcerated populations are especially vulnerable to communicable disease spread, including SARS-CoV-2, the virus responsible for COVID-19 (*1*–*3*). Outbreaks in correctional facilities have been linked to outbreaks and disease spread in the wider community (*4*,*5*). Although incarcerated persons and correctional staff were recommended as a priority group to receive vaccination (*6*), reported willingness among employees and incarcerated persons to receive COVID-19 vaccines was lower than among the general population (*7*). Thus, outbreaks in prisons present a valuable opportunity to assess vaccine effectiveness in a real-world, high-risk environment.

## Outbreak and Conditions

**Figure 1:**
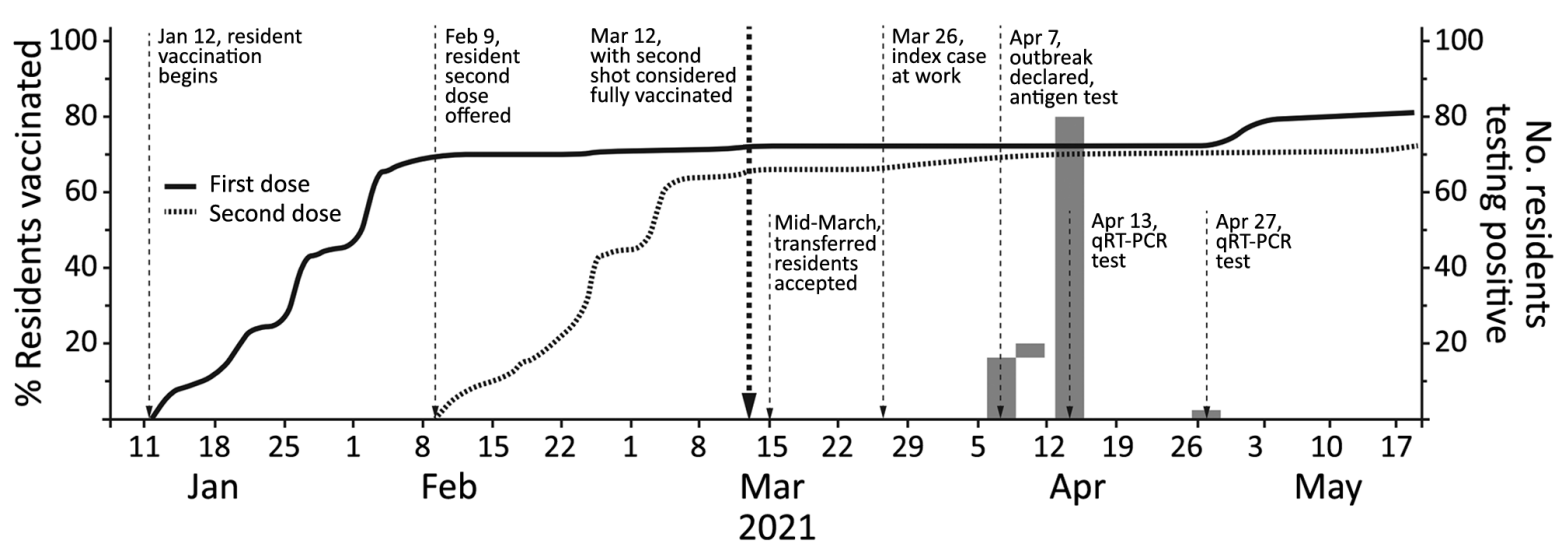
Timeline of SARS-CoV-2 vaccination rollout for incarcerated persons at a correctional facility in rural southwest Virginia included in analysis of vaccine effectiveness during a facility outbreak, April 2021.

On April 7, 2021, a COVID-19 outbreak in a men’s correctional facility in southwest Virginia was reported to the local branch of the Virginia Health Department (VDH) (Figure 1). Before this outbreak, this facility reported 46 employees and 2 residents (persons who were incarcerated) tested positive for SARS-CoV-2 during June 28, 2020–March 20, 2021, as well as 3 additional positive tests among employees during March 21–April 3, 2021. VDH was notified of 15 residents testing positive by rapid antigen test (BinaxNOW; Abbott, https://www.abbott.com), on April 7, followed by 4 more cases confirmed April 8–9, for a total of 19 positive antigen test results among 46 total residents who were tested because of symptoms or contact with a symptomatic or positive person (Figure 1).

On April 13 and April 27, 2021, employees and residents of the correctional facility were offered (with the option to decline) a quantitative reverse transcription PCR (qRT-PCR)–based test for SARS-CoV-2 using nasopharyngeal specimens as part of the VDH outbreak response. We conducted whole-genome sequencing and single-amplicon analyses on all positive samples to identify the source of the outbreak and the virus variants. At the time of the outbreak, the facility housed 865 residents (within the facility’s capacity) and had 300 employees. COVID-19 vaccines had been offered to both employees and residents, who were eligible as a priority population in early 2021. Health department officials stated that a total of 668 residents (77.2%) and 116 employees (38.7%) were fully vaccinated at the time of the outbreak. All vaccinated residents had received the Moderna vaccine (mRNA-1273). All cases associated with the outbreak were identified within 5 months of the initial vaccination rollout for residents and less than 3 months after their first opportunity to be fully vaccinated.

Mitigation measures for employees have been in place at this facility since April 2020 and include the use of mandatory face coverings and screening for symptoms 3 separate times before accessing residential areas. Employees were required to wear N95 masks when working with a positive resident, N95 or surgical masks when working with residents placed in quarantine, and cloth masks when working with persons with no known exposures or cases. Mitigation measures in place for the residents include daily medical screening, zone separations (e.g., quarantine and isolation units), cloth face coverings, limited transfers between housing units, discontinued cafeteria-style meals, and increased cleaning in living quarters. Routine surveillance testing was not conducted before this outbreak; however, once a case was identified, all employee close contacts were excluded from work and required to test negative before returning. Residents in close contact were moved to a yellow zone (e.g., quarantine precautions) with increased screening; they were tested if symptoms developed and before they returned to the green zone (e.g., general precautions). In accordance with VDH recommendations, facilitywide qRT-PCR testing was conducted in November 2020 (2 residents tested positive with no subsequent transmission) and in February 2021 (0 residents tested positive).

In the April 2021 outbreak, the initial patient, a resident, was tested because he reported symptoms during the daily screening; the result was positive. Everyone in that housing unit was then tested, and additional positives were identified. In the next week, additional symptomatic residents in that unit were tested and were positive. At the time of the outbreak, all vaccinated residents had received Moderna vaccines (mRNA-1273; https://www.modernatx.com). Vaccinated employees may have received either Moderna or Pfizer-BioNTech (BNT162b2; https://www.pfizer.com). The use of N95 masks was recommended in all units with positive cases or exposures during the April 2021 outbreak once it was identified.

The VDH April 2021 outbreak response included a site visit from an infection preventionist. VDH epidemiology staff identified multiple failures contributing to the outbreak, including improper mask use by some employees and a screening failure of a mildly symptomatic employee. VDH determined that 2 unvaccinated employees had come to work while infectious; their samples were collected on March 28 and March 31, and both tested positive for SARS-CoV-2. Sequence analyses identified mutations confirming that all cases resulted from these 2 employees. The VDH investigation established that the earliest day the index-case employee worked while infectious was March 26.

## Methods

We linked vaccine, demographic, and laboratory information using deidentified, automatically generated, unique identifiers that were verified by VDH staff with access to identifiable information. We excluded persons from participating if information was incomplete or if staff were unable to verifiably link subject information to their laboratory results in the data. Available demographic information was age, sex, and race.

We defined being fully vaccinated as having received a second vaccination by March 12, which was >14 days before the initial infectious employee returned to work on March 26 following out-of-state travel. We defined being partially vaccinated as having received 1 dose by March 12. We defined unvaccinated as receiving the vaccine on or after March 13 or not having received a vaccine at the time of this analysis. We defined SARS-CoV-2 infection as testing positive by qRT-PCR (*8*) or the rapid BinaxNOW antigen test.

Our qRT-PCR test received Emergency Use Authorization status from the FDA. The test’s technical development, as well as the assays that determine its specificity, sensitivity, and validation, have been described previously (*8*). Of note, specimens collected for analysis are transported in a specially formulated transport media containing chaotropic agents and stabilizers that ensure the quality of the sample is maintained for up to 10 days, even in the absence of a cold chain (*8*). The quantitative aspect of the assay helps determine the viral load of the sample: each plate includes dilution standard curves with known number of copies for each nucleocapsid protein (*N*) and ribonuclease P/MRP subunit P30 (*RPP30*) gene (range 5–500,000 copies/reaction for each gene) for which a specific cycle threshold (Ct) is generated and clinical results are extrapolated to estimate viral copies. Each person’s sample had 2 replicates qRT-PCR Ct values generated for each gene (*N*, envelope [*E*], spike [*S*], and housekeeping *RPP30*) (*8*). We used the mean Ct of each gene to generate the raw Ct value for comparison to the reference curve. In rare instances, a single value was used if one of the replicates did not amplify for a gene after a 45-cycle amplification limit; out of 92 cases, we did this for *N* gene for 3 cases, for 4 cases for *E* gene, and for 3 cases for *S* gene. We normalized Ct values by multiplying raw Cts by a correction factor defined as the ratio of the sample’s mean Ct value for *RPP30* over the mean *RPP30* for all samples in the plate (*9*).

We obtained genome data on all qRT-PCR positive samples using next-generation sequencing and amplicon sequencing approaches. In brief, we sequenced genomic libraries on a MiSeq system (Illumina, https://www.illumina.com) and aligned FASTQ reads to the Wuhan-Hu-1 sequence (GenBank accession no. NC_045512.2). In all cases, Illumina coverage of the consensus sequence was >99.4% of the reference genome. For single-amplicon sequencing, we performed targeted amplification using 13 sets of primers designed in-house for SARS-CoV-2 (*10*). We confirmed mutations by Sanger sequencing.

We limited statistical analyses to the residential population. We excluded data for employees whose demographic data were unavailable or whose tests were conducted outside the correctional facility. First, we assessed disparities in vaccination status across age and race at the time of the outbreak. Second, we estimated the relative risk (RR) of a SARS-CoV-2 infection regardless of symptoms when comparing unvaccinated to vaccinated residents using Poisson regression with robust variance estimates, with and without adjustment for age and race. We calculated vaccine effectiveness (VE) using the estimated RR from these models in which the numerator is the risk among unvaccinated and the denominator is the risk among vaccinated (VE = 1 − 1/RR). Third, we investigated differences in infection risk by age and race, stratifying this by vaccine status in Poisson regression with robust variance estimates. Last, we compared normalized Ct values between vaccinated and unvaccinated cases, with and without adjustment for age and race, using linear regression with robust variance estimates.

## Results

Test results from qRT-PCR were available for 854 (98.7%) of the 865 male residents at the facility at the time of the outbreak; 14 (1.6%) results were reported to be indeterminate (*7*). The timeline for testing was as follows: 823 residents were tested on April 13; of those, 732 were retested on April 27, and another 31 were tested on that date for the first time. Of the 19 residents who rapid-tested positive during April 7–9, a total of 17 were tested by qRT-PCR on April 13, and 16 yielded positive results. Although no deaths were reported as a result of this outbreak, an unvaccinated 64-year-old White male resident was hospitalized. Sequence analysis found that all qRT-PCR positive cases were linked to 1 of 2 index cases (employees); 96.2% of samples were identified as the B.1.1.7α (UK) variant of SARS-CoV-2. Of those, 3.8% did not pass the quality control metrics required to assign a variant.

Among the 854 residents with test results (35.8% White, 63.7% African American; mean age 40.4 years, range 18.8–86.0 years), 566 (66.3%) were considered fully vaccinated (76.1% White and 60.7% African American; mean age 42.4 years), 49 (5.7%) were partially vaccinated, and 239 (28.0%) were unvaccinated (mean age 35.4 years) by March 26. For both unvaccinated and vaccinated residents, the mean age was higher among positive cases compared with negative cases (38.5 vs. 34.3 years for unvaccinated; 43.2 vs. 42.3 years for fully vaccinated). Indeterminate qRT-PCR results were reported for 9 (1.6%) fully vaccinated and 5 (2.1%) unvaccinated residents and were excluded from the analyses. For those considered fully vaccinated, the median time from the second shot to March 28 was 33 days (26–46 days) for cases and 32 days (17–47 days) for noncases.

Among the 840 with definitive qRT-PCR test results, 19/557 (3.4%) fully vaccinated, 3/49 (6.1%) partially vaccinated, and 62/234 (26.5%) unvaccinated residents tested positive for SARS-CoV-2 infection (Figure 1). Partially vaccinated residents were excluded from subsequent analysis because of the small number. In unadjusted Poisson regression, unvaccinated residents were 7.8 (95% CI 4.8–12.7; p<0.001) times more likely to test positive during the April outbreak than were fully vaccinated residents (Table). This result corresponds to an unadjusted vaccine effectiveness VE of 87.1% (95% CI 79.0%–92.1%). Adjusting for age and race, unvaccinated residents were 8.8 (95% CI 5.2–14.9) times more likely to test positive compared with fully vaccinated residents, corresponding to an adjusted VE of 88.7% (95% CI 80.9%–93.3%; p<0.001).

**Table:** Relative risk of SARS-CoV-2 infection among incarcerated persons during an outbreak at a men’s correctional facility in rural Virginia, April 2021*

Characteristic	Unadjusted			Adjusted	
	No. persons	RR (95% CI)		No. persons	RR (95% CI)
Combined					
Unvaccinated *vs*. fully vaccinated	791	7.77 (4.75–12.69)†		787	8.82 (5.23–14.90)†
Age, 1 y increase	791	0.99 (0.98–1.01)		787	1.03 (1.01–1.05)‡
Race. Black *vs.* White	787	1.96 (1.19–3.258)‡		787	1.51 (0.91–2.49)
Stratified by vaccine status					
Fully vaccinated					
Age, 1 y increase	557	1.01 (0.97–1.05)		554	1.01 (0.97–1.05)
Race, Black vs. White	554	1.21 (0.48–3.02)		554	1.24 (0.49–3.14)
Unvaccinated					
Age, 1 y increase	234	1.03 (1.01–1.05)‡		233	1.04 (1.01–1.06)‡
Race, Black vs. White	233	1.40 (0.79–2.49)		233	1.70 (0.92–3.14)

*Relative risk calculated using Poisson regression. RR, relative risk. †p<0.001. ‡p<0.01.

When we adjusted for vaccine status, age, and race, older age was more statistically significant with testing positive (a 1-year increase in age RR 1.03, 95% CI 1.01–1.05; p = 0.005), and race was not statistically significant with testing positive (African American vs. White RR 1.51, 95% CI 0.91–2.49; p = 0.109). When we stratified by vaccine status and adjusted by race, age was associated only with testing positive among unvaccinated persons (RR 1.04, 95% CI 1.01–1.06; p = 0.001); we observed no association between testing positive and age among vaccinated residents (RR 1.01, 95% CI 0.97–1.05; p = 0.715).

**Figure 2:**
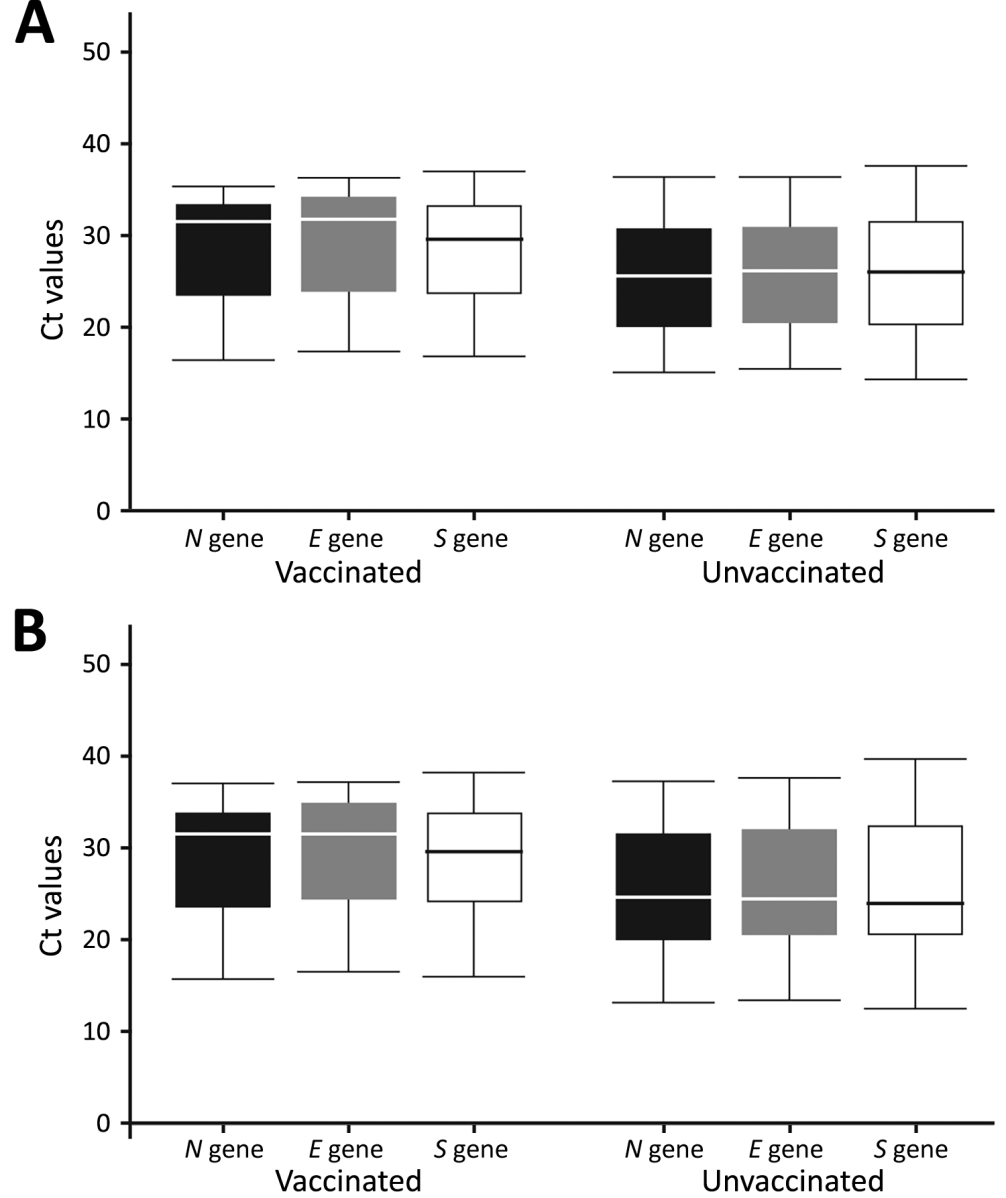
Raw (A) and normalized (B) Ct values for SARS-CoV-2 *N*, *E*, and *S* genes in samples collected from fully vaccinated and unvaccinated infected incarcerated persons during a facility outbreak, April 2021. The midline of the boxes represent the medians of the observations, the bottoms represents the first quartile, and the tops represent the third quartile; whiskers represent the minimum and maximum observations. In unadjusted linear regression comparing fully vaccinated to unvaccinated infected persons, only the E gene had statistically significantly different raw Ct values (p<0.05). All 3 genes had statistically significantly different normalized Ct values. Ct, cycle threshold; *E*, envelope gene; *N*, nucleocapsid gene; *S*, spike gene.

Among the infected, the unvaccinated showed lower raw and normalized Ct values compared to the vaccinated, indicating a higher viral load among the unvaccinated for all 3 genes (*N*, *E*, and *S*) (Figure 2). Normalized Ct values were statistically significantly lower among the unvaccinated compared with the vaccinated when we adjusted for age and race in linear regression for all 3 genes: for *N*, Ct value difference = 4.06 (95% CI 0.69–7.42; p = 0.019); for *E*, Ct value difference = 4.22 (95% CI 1.00–7.44; p = 0.011); for *S*, Ct value difference 3.90 (95% CI 0.49–7.32; p = 0.026).

## Discussion

This study shows that the Moderna vaccine for COVID-19 is highly effective at preventing SARS-CoV-2 infection among high-risk incarcerated persons, reducing infections by 87% when comparing vaccinated to unvaccinated persons. Although we observed some breakthrough cases, severe COVID-19 was uncommon (1 hospitalization) during this outbreak, and VE remained high and in agreement with reports in other settings in which the B.1.1.7α (UK) variant was prevalent (*11*). Results showed that younger age and Black or African American race were associated with lower vaccine uptake compared to older and non-Hispanic White populations; these differences were observed in the general US population as well (*12*).

Of note, and largely because of the rapid public health response to this outbreak, some case information was not collected; thus, this study has several limitations. First, we did not collect information on comorbidities, symptoms, stage of infection at the time of the test, prior infection and potential associated immunity, and ethnicity. Second, there was no information on contact patterns and corresponding risk for exposure. Third, 14 qRT-PCR results were indeterminate and excluded from the analysis. Fourth, some cases early in the outbreak (during March 28–April 13) could have been missed and misclassified as negative based on the initial qRT-PCR testing on April 13. However, we expect any such misclassification to be nondifferential with respect to vaccination status, resulting in an attenuation toward the null, and our results would be an underestimation of the true vaccine effectiveness. Finally, information regarding why some persons chose not to receive the vaccine or were not tested by qRT-PCR on either April 13 or April 27 was unavailable, which could bias the estimates of vaccine effectiveness if patients not being vaccinated or tested correlated with prior infection (and associated immunity). Fortunately, almost all residents (99%) were qRT-PCR tested on April 13, April 27, or both, and information was verified whenever possible. Therefore, any missing data or residual errors likely have minimal effect on estimates. Of note, most residents in the study had been housed long-term in this facility because no transfers into the facility occurred until mid-March 2021 and no prior COVID-19 outbreaks among residents were reported before April 2021. Thus, immunity from prior infections is likely negligible.

Methods from this study can be applied to similar settings. As new variants emerge and immunity may decrease (*13*), continued VE monitoring is needed to ensure public health strategies are well-informed and effective, especially in high-risk settings such as correctional facilities (*14*). High vaccination coverage among incarcerated persons, correctional facility staff, and the general population is critical to alleviate the challenges of the ongoing pandemic. In addition, sentinel or universal testing in correctional facilities may be necessary to prevent outbreaks (*15*), along with maintained compliance of other mitigation measures, such as masking and screening, proper ventilation, and vaccination boosters.
